# Association of the COVID-19 Pandemic With Unstable and/or Unsafe Living Situations and Intimate Partner Violence Among Pregnant Individuals

**DOI:** 10.1001/jamanetworkopen.2023.0172

**Published:** 2023-02-22

**Authors:** Lyndsay A. Avalos, G. Thomas Ray, Stacey E. Alexeeff, Sara R. Adams, Monique B. Does, Carey Watson, Kelly C. Young-Wolff

**Affiliations:** 1 Division of Research, Kaiser Permanente Northern California, Oakland; 2 Obstetrics and Gynecology, Kaiser Permanente, Antioch Medical Center, Antioch, California; 3 Department of Psychiatry and Behavioral Sciences, University of California, San Francisco

## Abstract

**Importance:**

The social, behavioral, and economic consequences of the COVID-19 pandemic may be associated with unstable and/or unsafe living situations and intimate partner violence (IPV) among pregnant individuals.

**Objective:**

To investigate trends in unstable and/or unsafe living situations and IPV among pregnant individuals prior to and during the COVID-19 pandemic.

**Design, Setting, and Participants:**

A cross-sectional population-based interrupted time-series analysis was conducted among Kaiser Permanente Northern California members who were pregnant and screened for unstable and/or unsafe living situation and IPV as part of standard prenatal care between January 1, 2019, and December 31, 2020.

**Exposures:**

COVID-19 pandemic (prepandemic period: January 1, 2019, to March 31, 2020; during pandemic period: April 1 to December 31, 2020).

**Main Outcomes and Measures:**

The 2 outcomes were unstable and/or unsafe living situations and IPV. Data were extracted from electronic health records. Interrupted time-series models were fit and adjusted for age and race and ethnicity.

**Results:**

The study sample included 77 310 pregnancies (74 663 individuals); 27.4% of the individuals were Asian or Pacific Islander, 6.5% were Black, 29.0% were Hispanic, 32.3% were non-Hispanic White, and 4.8% were other/unknown/multiracial, with a mean (SD) age of 30.9 (5.3) years. Across the 24-month study period there was an increasing trend in the standardized rate of unsafe and/or unstable living situations (2.2%; rate ratio [RR], 1.022; 95% CI, 1.016-1.029 per month) and IPV (4.9%; RR, 1.049; 95% CI, 1.021-1.078 per month). The ITS model indicated a 38% increase (RR, 1.38; 95% CI, 1.13-1.69) in the first month of the pandemic for unsafe and/or unstable living situation, with a return to the overall trend afterward for the study period. For IPV, the interrupted time-series model suggested an increase of 101% (RR, 2.01; 95% CI, 1.20-3.37) in the first 2 months of the pandemic.

**Conclusions and Relevance:**

This cross-sectional study noted an overall increase in unstable and/or unsafe living situations and IPV over the 24-month period, with a temporary increase associated with the COVID-19 pandemic. It may be useful for emergency response plans to include IPV safeguards for future pandemics. These findings suggest the need for prenatal screening for unsafe and/or unstable living situations and IPV coupled with referral to appropriate support services and preventive interventions.

## Key Points

**Question:**

Is there an association of the COVID-19 pandemic with trends in unstable and/or unsafe living situations and intimate partner violence (IPV) among pregnant individuals?

**Findings:**

In this cross-sectional analysis of 77 310 pregnancies (74 663 individuals) occurring between January 1, 2019, and December 31, 2020, there was a 38% increase in unstable and/or unsafe living situations in the first month of the pandemic and a 101% increase in IPV in the first 2 months of the pandemic. However, overall increasing trends in unstable and/or unsafe living situations and IPV over the study period were not affected by the pandemic.

**Meaning:**

The findings of this study suggest that IPV safeguards are needed in pandemic emergency response plans.

## Teaser

This cross-sectional study compares the rates of unstable and/or unsafe living situations and intimate partner violence in individuals who were pregnant before vs during the COVID-19 pandemic.

## Introduction

Intimate partner violence (IPV) is one of the most common forms of violence against women and a major public health concern. Defined as physical, sexual, psychological, and/or economic abuse that occurs by an intimate partner,^1^ nearly 25% of women experience IPV at some point in their life^2^ and it is most prevalent among women of reproductive age. Intimate partner violence during pregnancy is associated with adverse effects on maternal and child health, including preterm birth,^3,4^ low birth weight,^4^ neonatal hospitalization,^4^ and prenatal and postpartum mental health problems.^5,6^

The social, behavioral, and economic consequences of the COVID-19 pandemic and shelter-in-place orders enacted to slow the spread of the virus may have contributed to IPV and unstable and/or unsafe living situations. Data suggest a worldwide increase in the prevalence of IPV during the COVID-19 pandemic, with the United Nations describing it as a shadow pandemic within the pandemic.^7,8,9^ In the US, at the beginning of the pandemic, several cities experienced an increase in domestic violence police service calls,^10^ and trauma center IPV-related injury admissions increased.^2^ Despite pregnancy being a period with increased susceptibility to adverse health effects for both the mother and offspring, the consequences of the COVID-19 pandemic on IPV and unstable and/or unsafe living situations in pregnant individuals have been less studied. To address this gap and better understand any association between the COVID-19 pandemic and pregnancies, we used data from the Kaiser Permanente Northern California (KPNC) large integrated health care delivery system with universal screening for IPV and unstable and/or unsafe living situations. Based on anecdotal reports^11^ and existing research on IPV,^7,8,9^ we hypothesized that IPV and unstable and/or unsafe living situations increased during the COVID-19 pandemic.

## Methods

### Setting and Study Population

Kaiser Permanente Northern California is an integrated health care delivery system with 21 hospital-based medical centers serving approximately 4.4 million racially and sociodemographically diverse patients who are representative of Northern California.^12^ On March 4, 2020, following the state’s first COVID-19 death, California’s governor declared a state of emergency.^13^

Using electronic health record data, we identified all IPV screening questionnaires completed during standard prenatal care in KPNC between January 1, 2019, and December 31, 2020. The screening questionnaire is typically completed at entrance to prenatal care (approximately 8 weeks’ gestation). For each pregnancy, we extracted age at pregnancy onset (imputed to be 14 days after the last reported menstrual period) and self-reported race and ethnicity from the electronic health record data to account for differences by these factors when modeling. The KPNC Institutional Review Board approved the study and waived informed consent. Study procedures meet Health Insurance Portability and Accountability Act requirements and the 42 CFR Part 2 regarding medical records. On enrollment in the health plan, all KPNC members are informed that their data may be used for research. Data were not deidentified. The study followed the Strengthening the Reporting of Observational Studies in Epidemiology (STROBE) reporting guideline for cross-sectional studies.

### Outcomes

The following 2 questions from the prenatal IPV screening questionnaire were used to define the 2 outcomes unsafe and/or unstable living situation and IPV: “Is your living situation unsafe and/or unstable?” “Are you in a relationship with a person who threatens or physically hurts you?” Participants could answer yes or no to each question.

### Exposure

Our goal was to evaluate the association of the COVID-19 pandemic with unsafe and/or unstable living situations and IPV. Therefore, we treated the COVID-19 pandemic as the exposure of interest. Pregnancies were categorized as occurring either before the pandemic (questionnaire completed between January 1, 2019, and March 31, 2020) or during the pandemic (questionnaire completed between April 1 and December 31, 2020).

### Statistical Analysis

For each outcome, strata were created by calendar month, age (<25, 25 to <35, ≥35 years) and self-reported race and ethnicity (Asian or Pacific Islander, Hispanic, non-Hispanic Black, non-Hispanic White, and unknown or other [American Indian, Alaskan Native, multiracial]). The categories were based on self-report and ascertained from the electronic health record. We combined other/unknown and multiracial into 1 category. For each of the 2 outcomes, we computed the rate of respondents answering yes per 1000 pregnancies during each calendar month of the study, standardized to the year 2020 age and race and ethnicity distribution of the cohort. These standardized rates were used to plot rates over time.

For each outcome, we fit an interrupted time-series (ITS) model to the monthly data using negative binomial regression. The input data set consisted of 1 record per month from January 1, 2019, to December 31, 2020, for each age and race and ethnicity group (24 months ×3 age groups ×5 race and ethnicity groups = 360 total records). The dependent variable was the monthly count of the number of individuals answering yes to the question, the offset variable was the log of the number of pregnant individuals responding to the question, and the covariables included age and race and ethnicity. We first analyzed the rate during the pre-COVID-19 period to determine whether it was stable, increasing, or decreasing over time. In preliminary analyses of the change during the COVID-19 pandemic, we conducted visual inspection of the rates over time and considered the potential for different types of changes during the COVID-19 pandemic defined in ITS analyses: a level change, a slope change, a change in both the level and slope, or an increase (temporary level change).^14^ These preliminary analyses indicated that the model for a temporary level change would be most appropriate. We also conducted preliminary analyses to assess whether the trend following the temporary level change differed from the prepandemic trend; these preliminary analyses found that the additional continuous time variable capturing the change in the trend (slope) was nonsignificant for both outcomes and was therefore excluded from the final models. Thus, our final ITS models included a continuous time variable to capture the expected trend (slope) in the percentage of individuals answering yes to this question over time and a dichotomous variable that indicated the COVID-19 temporary level change period, which was 1 month (April 2020) for unstable and/or unsafe living situation and 2 months (April and May 2020) for IPV. We report the rate ratio and corresponding 95% CIs for the trend over time and the increase (temporary level change period) for each outcome. Analyses were conducted in SAS, version 9.4 (SAS Institute Inc). Two-sided *P* values <.05 were considered statistically significant.

## Results

The study sample included a total of 77 310 pregnancies (74 663 individuals); 27.4% of the individuals were Asian or Pacific Islander, 6.5% were Black, 29.0% were Hispanic, 32.3% were non-Hispanic White, and 4.8% were other/unknown/multiracial, with a mean (SD) age of 30.9 (5.3) years (Table). During the entire study period, 2.6% of individuals reported an unsafe and/or unstable living situation and 0.2% reported IPV. When comparing pregnancies during the pandemic with those before the pandemic, differences in demographic characteristics, such as maternal age, race and ethnicity, and gestational age at screening, were small (from 0.3% to 2.1% for race and ethnicity, and 0.3-week difference in mean gestational age at screening) (Table).

**Table.** Pregnancies Screened for Unsafe and/or Unstable Living Situations and Intimate Partner Violence, 2019 to 2020, Kaiser Permanente Northern California

Characteristic	All pregnancies (N = 77 310), No. (%)	Timing of response to screening questions, No. (%)^a^	
		Prior to COVID-19 (n = 52 719)	During COVID-19 (n = 24 591)
Age, mean (SD), y	30.9 (5.3)	30.9 (5.3)	30.9 (5.2)
Race and ethnicity			
Asian or Pacific Islander	21 212 (27.4)	14 423 (27.4)	6789 (27.6)
Black	4997 (6.5)	3475 (6.6)	1522 (6.2)
Hispanic	22 442 (29.0)	15 004 (28.5)	7438 (30.2)
Non-Hispanic White	24 967 (32.3)	17 375 (33.0)	7592 (30.9)
Unknown/other^b^	3692 (4.8)	2442 (4.6)	1250 (5.1)
Weeks from pregnancy start to pregnancy circumstances questionnaire response^c^			
Mean (SD)	8.1 (4.7)	8.2 (4.8)	7.9 (4.5)
Median (IQR)	6.9 (5.7-8.7)	7.0 (5.9-8.9)	6.6 (5.6-8.6)

^a^ Prepandemic was January 1, 2019, to March 31, 2020, and during pandemic was April 1 to December 31, 2020.

^b^ Other race and ethnicity includes American Indian, Alaskan Native, and multiracial.

^c^ Pregnancy start was imputed to be 14 days after the last reported menstrual period.

**Figure 1.**
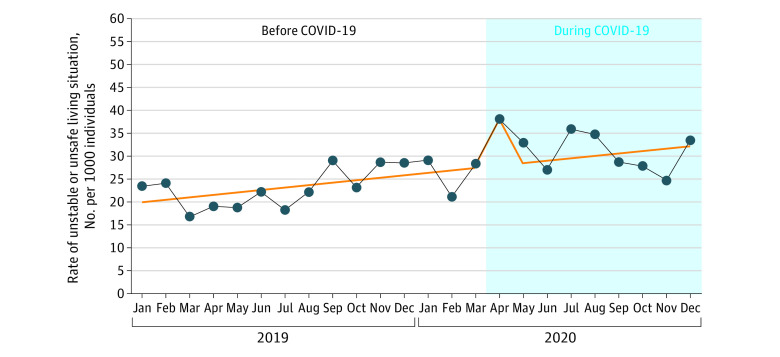
Rate per 1000 Pregnancies for Unstable and/or Unsafe Living Situations Before and During the COVID-19 Pandemic

The standardized rates of unsafe and/or unstable living situations ranged from 17 per 1000 to 29 per 1000 pregnancies per month before the pandemic, reaching a high of 38 per 1000 in April 2020 at the start of the pandemic and ranging from 25 per 1000 to 36 per 1000 pregnancies per month for the remaining months of the pandemic study period (May-December 2020). The ITS models found a steady relative increase (trend) in the rate of unsafe and/or unstable living situations of 2.2% per month (rate ratio, 1.022; 95% CI, 1.016-1.029) over the 24-month study period, and an increase of 38% (rate ratio, 1.38; 95% CI, 1.13-1.69) in the first month of the pandemic (Figure 1). There was no significant difference in the overall trend before and during the pandemic.

**Figure 2.**
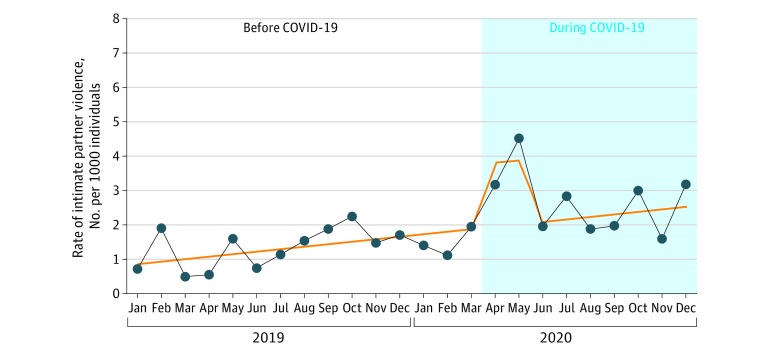
Rate per 1000 Pregnancies for Intimate Partner Violence Before and During the COVID-19 Pandemic

The standardized rates of IPV ranged from 0.49 per 1000 to 2.24 per 1000 pregnancies per month before the pandemic, reaching a high of 4.51 per 1000 in May 2020 and ranging from 1.58 per 1000 to 3.17 per 1000 per month for the remaining months of the pandemic study period (June-December 2020). The ITS models found a steady relative increase in the rate of IPV of 4.9% per month (rate ratio, 1.049; 95% CI, 1.021-1.078) over the 24-month study period, and an increase of 101% (rate ratio, 2.01; 95% CI, 1.20%-3.37%) in the first 2 months of the pandemic (Figure 2). There was no significant difference in the overall trend before and during the pandemic.

## Discussion

In this study of 77 310 pregnancies, the beginning of the COVID-19 pandemic was associated with a significant temporary increase in the rates of unstable and/or unsafe living situations and IPV during pregnancy. In addition, we documented an increasing trend in unstable and/or unsafe living situations and IPV over the 24-month study period. The temporary increase corresponds with the height of the shelter-in-place advisories implemented to control the spread of the virus.^15^ The mandatory lockdowns and shelter-in-place policies may have forced isolation at home with an abusive partner. The psychological toll of the pandemic may have exacerbated preexisting mental health or substance use conditions among perpetrators, leading to more violent or controlling behaviors. In addition, the pandemic led to the highest recorded US unemployment rate,^16^ leading to high rates of economic insecurity with potential for unstable and/or unsafe living situations. Economic insecurity is associated with IPV,^17^ highlighting the importance of economic security for violence prevention. To our knowledge, this is the first study using screening data as part of standard prenatal care to document an increase in unstable and/or unsafe living situations and IPV associated with the COVID-19 pandemic as well as a steady increase in the prevalence of these factors in recent years in the US.

The increase in unstable and/or unsafe living situations and IPV during pregnancy early in the COVID-19 pandemic documented in our study is similar to the increase in domestic violence police service calls in 14 large US metropolitan cities or areas.^10^ While it is not clear what led to the increase being characteristically temporary, increasing evidence suggests that cash transfer programs decrease IPV.^18^ The return to the baseline trend and the end of the increase coincided with the release of stimulus checks under the Coronavirus Aid, Relief, and Economic Security Act (CARES Act)^19^ as well as a relaxing of the shelter-in-place orders,^20^ which eased isolation and provided financial relief.^21^ Thus, improved access to financial support may have led to decreases in both unsafe and/or unstable living situations and IPV.

Findings from our study also replicate data documenting increases in IPV after natural disasters and public health emergencies.^22,23^ This pattern exemplifies the need for prenatal IPV screening during public health emergencies and enhancing resources for pregnant individuals experiencing IPV. Interventions (eg, shelter-in-place policies) to address public health crises should not cause other harms. Therefore, it is also critical to implement IPV safeguards into emergency response plans. During public health emergencies, the use of technology for screening may be useful when access to in-person services may be limited.

The near doubling in the rate of IPV over the 24-month period signifies the ongoing need for prevention interventions. Routine screening for IPV in prenatal care is recommended by the American College of Obstetrics and Gynecology.^24^ In addition, health care systems and the medical community can respond to the increasing prevalence of IPV among pregnant individuals by offering trauma-informed support and counseling and providing prevention and referral resources.^25^

### Strengths and Limitations

This study had several strengths. Of note, the study addresses the limitations of previous research that used data from social media, internet, anecdotal evidence, helpline reports, or emergency department visits that were limited to the first 1 or 2 months of the pandemic.^26^ We used high-quality data ascertained through a screening program for IPV integrated into standard prenatal care. Interrupted time-series modeling is the reference standard method for assessing the association of COVID-19 with trends. While a recent systematic review documented a high prevalence of IPV during the pandemic worldwide,^27^our study is among the largest population-based studies evaluating trends in unstable and/or unsafe living situations and IPV among individuals who were pregnant during the COVID-19 pandemic. The study included a racially and ethnically and geographically diverse sample of more than 77 000 pregnancies with high generalizability. Thus, findings from this study provide a clearer understanding of the association between the pandemic, unsafe and/or unstable living situations, and IPV among an extremely vulnerable population.

This study has limitations. The analysis only included prenatal screening for unsafe and/or unstable living situations and IPV completed at the beginning of pregnancy (approximately 8 weeks’ gestation); thus, the standardized rates could be an underestimate. Findings may be associated with individuals being more likely to report these outcomes as opposed to actual increases. An unstable and/or unsafe living situation is somewhat ambiguous, and we were unable to differentiate whether results reflect increases in violence, fear of SARS-CoV-2 infection resulting from a high-risk domestic environment, or financial insecurity leading to an unstable housing situation. However, the patterns for the 2 outcomes were similar, with both resulting in an increasing trend over the 24-month period and a temporary increase at the beginning of the COVID-19 pandemic. While screening for unstable and/or unsafe living situations and IPV is supposed to be universal at KPNC, it was not documented for 29% of pregnancies, which may have impacted our findings. In addition, we were not able to capture more nuanced dimensions of control, such as social isolation or psychological abuse. Finally, this study included pregnant individuals with health insurance; thus, findings may underestimate the rates in uninsured/underinsured and disadvantaged populations.

## Conclusions

Intimate partner violence among individuals who are pregnant is a major public health problem, associated with multigenerational long-term negative health and mental health consequences. Findings from this study suggest a need to support those experiencing or at-risk for unsafe and/or unstable living situations and IPV, particularly at the beginning of pandemics. In addition, the increasing prevalence rates highlight the need for universal prenatal screening for both unstable and/or unsafe living situations and IPV with referral to appropriate support services.

## Notes

Supplement.
Data Sharing Statement
