# Supplementary material for: Association of the COVID-19 Pandemic With Unstable and/or Unsafe Living Situations and Intimate Partner Violence Among Pregnant Individuals
Source: JAMA Netw Open. 2023 Feb 22;6(2):e230172. doi: 10.1001/jamanetworkopen.2023.0172 (PMC9947729; doi:10.1001/jamanetworkopen.2023.0172)
Supplement: Supplement. — Data Sharing Statement [file jamanetwopen-e230172-s001.pdf]

Data Sharing Statement

Avalos. Association of the COVID-19 Pandemic With Unstable and/or Unsafe Living Situations and Intimate Partner Violence Among Pregnant Individuals. *JAMA Netw Open*. Published February 22, 2023. doi:10.1001/jamanetworkopen.2023.0172

Data
Data available: No
